# Socio-demographic Differences in Toxic Release Inventory Siting and Emissions in Metro Atlanta

**DOI:** 10.3390/ijerph13080747

**Published:** 2016-07-23

**Authors:** Ryan Johnson, Kim Ramsey-White, Christina H. Fuller

**Affiliations:** 1 St. Louis School of Medicine, Washington University, St. Louis, MO 63130, USA; johnson.r@wustl.edu; 2 Division of Epidemiology and Biostatistics, School of Public Health, Georgia State University, Atlanta, GA 30302, USA; kwhite@gsu.edu; 3 Division of Environmental Health, School of Public Health, Georgia State University, Atlanta, GA 30302, USA

**Keywords:** exposure disparities, Toxic Release Inventory, environmental regulation, environmental justice, socioeconomic status, minorities

## Abstract

Prior research has found that low socioeconomic status (SES) populations and minorities in some areas reside in communities with disproportionate exposure to hazardous chemicals. The objectives of this study were to evaluate the relevance of socio-demographic characteristics on the presence of Toxic Release Inventory (TRI) facilities, air releases, and prevalence and resolution of air quality complaints in the 20-county Atlanta Metropolitan Statistical Area (MSA). We found that there were 4.7% more minority residents in census tracts where TRI facilities were located. The odds ratio (OR) for the presence of a TRI facility was 0.89 (*p* < 0.01) for each 1% increase of females with a college degree and 2.4 (*p* < 0.01) for households with an income of $22,000–$55,000. The estimated reduction in the amount of chemicals emitted per release associated with population of females with a college degree was 18.53 pounds (*p* < 0.01). Complaints took longer to resolve in census tracts with higher Hispanic populations (OR = 1.031, 95% CI: 1.010–1.054). Overall, results indicate that SES and race/ethnicity are related to TRI facility siting, releases, and complaints in the Atlanta area. These findings have not been documented previously and suggest that lower SES and non-White communities may be disproportionately exposed.

## 1. Background

Exposure disparities are often founded upon inequities in zoning and planning driven by social factors such as socioeconomic status (SES) and race [1,2]. Low SES populations and minorities are often exposed to a disproportionate number of hazardous chemicals, including hydrogen fluoride, benzene, and formaldehyde [1]. Constant exposure to these harmful conditions results in negative health outcomes, stressed communities, and reduction in quality of life and neighborhood sustainability [3].

Many toxic air pollutants are known or suspected to cause serious health effects. The U.S. Environmental Protection Agency (EPA) designates 187 air pollutants as harmful to the environment and public health [4]. Examples of these pollutants include benzene, found in gasoline; hydrogen fluoride, emitted from coal burning power plants; and methylene chloride, used as a solvent and paint stripper by multiple industries [4]. There is a wide array of health effects of these chemicals, including cancer development, respiratory ailments, as well as neurological, reproductive, and developmental issues [5]. In addition to exposure through inhalation, some toxic air pollutants such as mercury can deposit onto soils or surface waters, where they can be taken up by plants and are eventually magnified up the food chain, a process called bioaccumulation [6].

The Toxic Release Inventory (TRI) Program was created in 1986 to track the management of certain toxic chemicals that are emitted by industrial facilities and may pose a threat to human health and the environment. TRI facilities include companies across a wide range of industries (including chemical, mining, paper, oil and gas) that produce more than 25,000 pounds or handle more than 10,000 pounds of a listed toxic chemical [4]. All U.S. facilities that meet reporting criteria must submit TRI data to the EPA each year [4].

Previous research has shown TRI facilities to be concentrated in urban areas with large minority populations [7,8]. Urban areas are of particular concern due to the large number of individuals within a small geographic area. Atlanta is the ninth-largest metropolitan statistical area (MSA) in the United States with a population of 5.3 million people [9]. Metro Atlanta has the second largest African American population in the United States and a steadily growing Hispanic population [9]. Due to Atlanta’s large minority population and the history of environmental injustice in the U.S., it is important to understand if and to what magnitude spatial disparities exist in regards to TRI site location, toxic chemical releases, and regulation. The purpose of this study was to provide a more complete and robust view of environmental justice in the South by examining inequality on multiple fronts. Specifically, we sought to determine whether the racial and socioeconomic composition of census tracts in Atlanta would be associated with the distribution of TRI facilities, the number of releases of hazardous air chemicals, and the amount of each release within the Atlanta MSA. Additionally, this study sought to examine if the number of complaints and the response to complaints differed between census tracts of different socioeconomic and racial compositions. Examination of TRI facility locations and air chemical releases can help to determine whether some populations in metropolitan Atlanta potentially have high exposure, and consequently, increased risk of adverse health outcomes. Examining differences in complaints to toxic chemical releases and the resolution of these complaints can help to improve regulation.

## 2. Methods

*Data Sources.* The data for this study were derived from the EPA’s TRI program (years 2006–2011); year 2000 demographic data from the U.S. Census Bureau Summary Files 1 and 3; and Georgia’s Environmental Protection Division (EPD) data on complaints to hazardous chemicals from 2006–2011.

We extracted year 2000 census-tract data from the U.S. Census Bureau Summary files and mapped TRI facility locations in the Atlanta MSA using ArcMAP 10.1 (Environmental Systems Research Institute, Redlands, CA, USA) and latitude-longitude coordinates. Geographical coordinates were used to match each TRI facility to its respective census tract. Data on air quality complaints to toxic chemicals were requested from Georgia EPD via an Open Records Request. Census tracts were derived from Georgia EPD’s complaint data by geocoding the pollution source address. Once all three data sets had the unique identification census tract number, they were merged. The unit of analysis for this study was the census tract. We used data from the 2000 census instead of the 2010 census because the latter used only a short form questionnaire, with long form data collected through the American Community Survey (ACS). For those data, which included our sociodemographic variables of interest, the ACS is not reliable at the census tract level [10].

*Geographic Coverage.* The Atlanta MSA as designated by the United States Office of Management and Budget is comprised of 20 counties: Barrow, Bartow, Carroll, Cherokee, Clayton, Cobb, Coweta, DeKalb, Douglas, Fayette, Forsyth, Fulton, Gwinnett, Hall, Henry, Newton, Paulding, Rockdale, Spalding, and Walton. There are 657 census tracts in the Atlanta MSA. 

*Socio-Demographic Data.* Socio-demographic characteristics within the Atlanta MSA were estimated at the census tract level. We had access to the following relevant variables: race/ethnicity (non-Hispanic White, non-Hispanic Black, Hispanic, and Asian), poverty (residents living below the national poverty level and residents on public assistance), educational attainment (female population older than 24 years with high school education or less, an undergraduate degree, or a graduate/professional degree), and vacant housing (percentage of houses vacant). Proportions (percentages) for each of these variables was estimated from the census data. Median household income was also estimated for each census tract. We used female educational attainment because it is an appropriate indicator of socioeconomic status in communities. Research indicates that female education is a fundamental factor determining the quality of life of women with resulting effects on the lives of children and families, and ultimately affects communities as a whole [11,12]. Female education also correlates with high levels of social and economic development.

*Toxic Release Inventory Data.* A self-administered reporting form collects TRI data, and data are reported by individual chemical or chemical category on a facility basis. The EPA examines these data for reporting errors and then compiles them into a centrally-managed database. Data is collected annually from facilities that meet the EPA’s criteria. These criteria are (1) employs 10 or more full-time equivalent employees; (2) manufactures or processes more than 25,000 lbs. of a TRI-listed chemical or otherwise uses more than 10,000 lbs. of a listed chemical in a given year; and/or (3) are TRI-Covered Industries of mining, utilities, manufacturing, merchant wholesalers, non-durable goods, wholesale electronic markets, and agents brokers publishing, and hazardous waste. This study was focused on air quality; therefore, we restricted our analysis to TRI facilities that emit air toxics. Outcomes of interest included TRI facility presence, the number of releases of hazardous air chemicals, and the amount of each release in pounds within a census tract. The number of releases were summed over the six year study period. The amount of each release was an average estimated by sum of releases over the six year study period divided by the number of releases.

*Complaint Data.* Georgia EPD provided data on all primary and secondary air quality complaints. Primary complaints were those that specifically cited air quality as the reason for the complaint. Secondary complaints were complaints made about another issue that resulted in an air quality impact. We pooled primary and secondary air quality complaints and treated them equally in our analysis. The complaint records included the address of the source, which we mapped to the corresponding 2000 census tract.

*Statistical Analysis.* We performed basic descriptive statistics on all socio-demographic, TRI, and complaint data. Logistic regression was used to assess whether race/ethnicity and socioeconomic variables were associated with TRI facility presence in a census tract. We applied linear regression models to examine the association between amount of air toxics released from TRI facilities in the census tract, the number of releases from TRI facilities, the amount of chemicals emitted per release, and socio-demographic variables at the census tract level. Bivariate models were created first and significant predictors of the outcome were put into multivariate models. Additionally, ordinal logistic regression was used to evaluate the association between the number of air quality complaints and time to resolution of complaints and the covariates (SES variables and race/ethnicity) at the census tract level. If necessary, variables were log transformed to achieve normal distributions. SAS version 9.3 (SAS Institute Inc., Cary, NC, USA) was used to perform statistical analyses.

## 3. Results

### 3.1. TRI Facility Distribution

**Figure 1:**
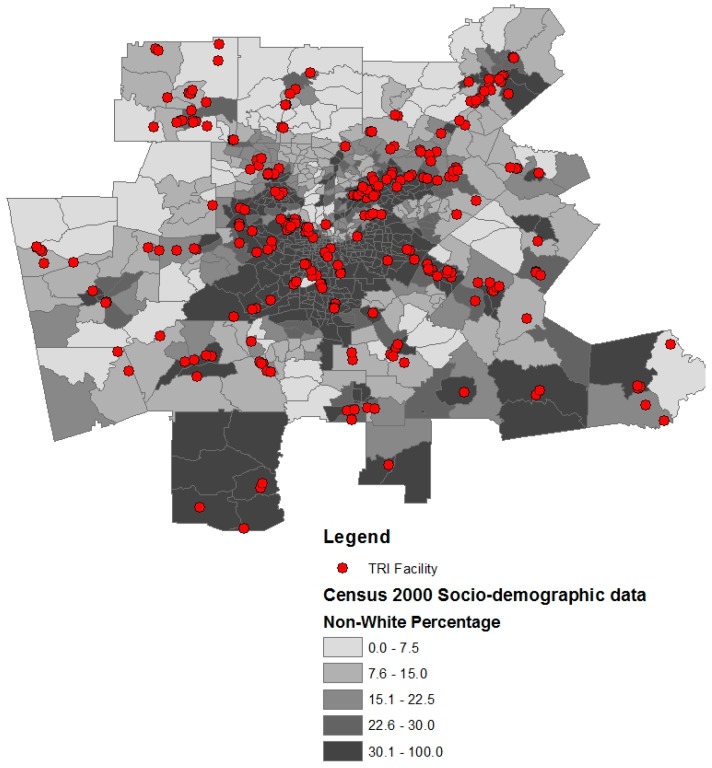
Metropolitan Atlanta Toxic Release Inventory (TRI) facility distribution and percent of population non-White.

**Figure 2:**
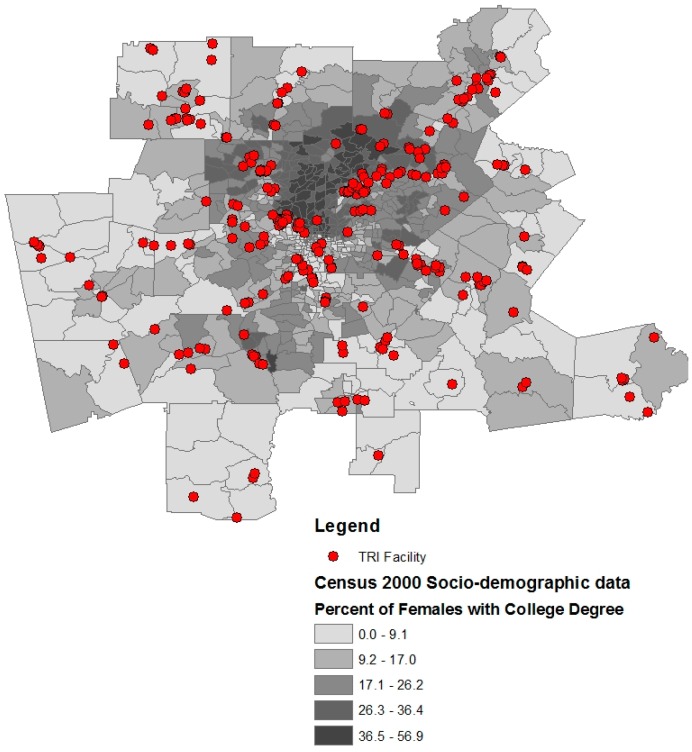
Metropolitan Atlanta Toxic Release Inventory (TRI) facility distribution and percent of females with undergraduate degree.

There are 676 census tracts in the Atlanta MSA, and the 922 TRI facilities are concentrated in 135 tracts. Table 1 and Table 2 show TRI data and demographic variables, respectively. Census tracts composed of a high percentage (>50%) of lower-middle class residents ($22,500–$55,000 household income) have more TRI facilities than more affluent tracts (Table 3). Specifically, 59.3% of TRI facilities are located in lower-middle class census tracts. In addition, census tracts composed of higher percentages of non-White individuals are more likely to have a TRI facility present in their census tract (Figure 1 and Table 3). Census tracts with a TRI facility have more Hispanics and African Americans than tracts without a TRI facility (Table 3). Female educational attainment was higher in census tracts without TRI facilities (Figure 2). Census tracts with TRI facilities had on average 4% fewer females with a college degree than census tracts with TRI Facilities (Table 3).

A multivariate logistic regression was fit to evaluate the association between the presence of a TRI facility and all SES and racial/ethnic composition variables that were significant in bivariate models (Table 4). The odds ratio (OR) for TRI facility presence in a census tract was 0.894 (*p* = 0.0002) for a unit increase in females with an undergraduate degree, 2.4 (*p* < 0.0001) for households with an income of $22,000–$55,000, and 0.985 (*p* = 0.0005) for a unit increase in percent Black population.

### 3.2. Toxic Air Chemical Releases

We employed multiple linear regression to examine the association between socio-demographic variables at the census tract level and (1) amount of air toxics released from TRI facilities in the census tract; (2) the number of releases from TRI facilities; and (3) and the amount of chemicals emitted per release. The total amount of chemicals released and amount of chemicals emitted per release were LOG10 transformed to mitigate violations of the linearity and normality assumptions. Furthermore, percent of population female with an undergraduate degree, percent of population Hispanic, and percent of population Black were also LOG10 transformed.

To evaluate the association between the amount of toxic air chemicals released, amount of chemicals emitted per release, and number of releases with SES and race/ethnicity, we fit separate multivariate linear regression models, which included significant variables from bivariate models. Percentage of females with an undergraduate degree was the only variable significantly associated with the amount of toxic air chemicals released (Table 5). The estimated difference in the amount of chemicals emitted associated with a one-percentage point difference corresponding to a 10-fold difference in percentage of population female with undergraduate degree—adjusting for median household income and percentage of population non-White is −18.53 pounds. Thus, on average, the lower female education census tracts with TRI facilities had more chemicals emitted than higher female education census tracts with TRI facilities (β = −0.1853, *p* = 0.009). Additionally, census tracts with lower percentage of female college graduates had more chemicals emitted per release (Table 5). The estimated difference in the amount of chemicals emitted per emission associated with a one-percentage point difference corresponding to a 10-fold difference in percent of population are females with undergraduate degree—adjusting for median household income and % of population non-White—is −18.0 pounds. Thus, on average, the lower female education census tracts in which TRI facilities were located had more chemicals emitted than higher female education census tracts with TRI facilities (β = −0.18, *p* = 0.004). 

The unadjusted Pearson’s correlation found that the number of releases from 2006–2011 had no statistically significant association with any socio-demographic characteristic. Furthermore, the multivariate analysis showed no statistically-significant association between any of the socio-demographic characteristics and the number of toxic air chemical releases in the years 2006–2011.

### 3.3. Complaints to Air Toxic Chemical Releases

**Figure 3:**
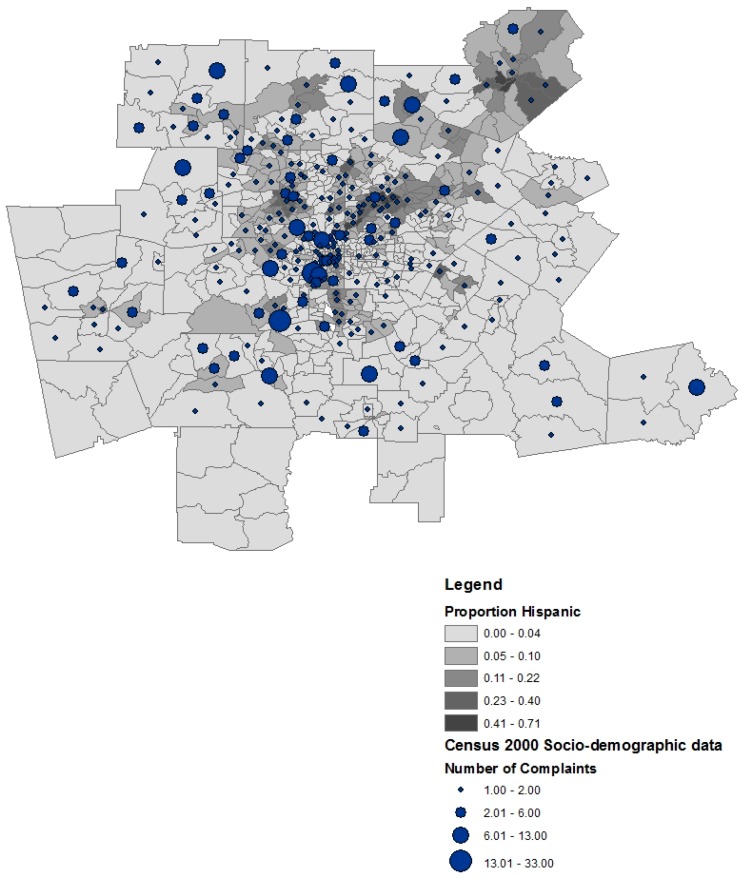
Number of complaints and proportion Hispanic in the Atlanta Metropolitan Area. (Note: complaints are assigned to the centroid of each tract).

To evaluate the association between the number of complaints to air toxic releases in census tract and socio-demographic variables, an ordinal logistic regression model was fitted on complaint categories. The complaint categories included no complaints, one complaint, and two or more complaints. Those census tracts that had multiple complaints regarding air toxics had 4.3% fewer non-White residents than the census tracts that had no complaints (β = 0.006, *p* = 0.009) (See Figure 3). The percent of population Black (OR = 0.989, *p* = 0.0006) and Asian (OR = 0.922, *p* = 0.0021) were significantly associated with the number of complaints to toxic chemical releases, controlling for median household income, TRI facility presence, percent of population female with an undergraduate degree, and percent Hispanic population (Table 6). Census tracts with higher percentages of Blacks and Asians were less likely to have reported complaints than other census tracts. On average, census tracts with a higher proportion female college graduates were less likely to report toxic chemical releases (OR = 0.957, *p* = 0.05). On average, the census tracts with multiple complaints had 2% fewer females with college degrees than census tracts with no complaints. Overall, female education and race/ethnicity seem to be the most influential regarding complaints to air toxics.

### 3.4. Resolution of Complaints to Air Toxic Releases

The variables significantly associated with the resolution time of complaints was presence of a TRI facility and Hispanic composition (Table 6). As the percentage of Hispanic residents increased in a census tract, the time it took to resolve an environmental complaint to air toxics increased (OR = 1.031, *p* = 0.01), controlling for TRI facility presence in the census tract, median household income, percent of population Black, and percent of population female with an undergraduate degree.

## 4. Discussion

Using year-2000 census data, we found evidence of socio-demographic disparities in the location of TRI facilities and chemical releases in the Atlanta MSA. Female education was shown to be a consistent predictor across the range of exposures evaluated. As the percentage of college graduates increased in a census tract, the odds of that census tract having a TRI facility decreased, controlling for median household income, percent Hispanic population, and percent Black population. In this same model, census tracts composed of a high percentage (>50%) of lower-middle class residents ($22,500–$55,000 household income) had more TRI facilities than more affluent tracts (borderline significance *p* = 0.05). Specifically, 59.3% of TRI facilities are located in lower–middle class census tracts. The results of descriptive analysis revealed higher percentages of non-White residents in census tracts that have TRI facilities compared to other census tracts; however, this association disappears after controlling for other variables. We also evaluated the association between SES and race/ethnicity and the number of TRI releases and amount of chemical releases in pounds in each census tract. We found a statistically-significant relationship whereby a census-tract level increase in the percentage of females with college degree was associated with a reduction in the amount of chemicals released. These results indicate the role that female educational attainment plays in whether a census tract will have a TRI facility and how chemicals are released as an indication of burden disparities for low-SES populations in the Atlanta MSA.

These results are similar to those previously reported in the environmental justice literature [7,13]. A cross-sectional study conducted by Mohai et al. [13] found that African Americans and respondents at lower female educational levels and lower income levels were significantly more likely to live within a mile of a polluting facility. Racial disparities were especially pronounced in metropolitan areas of the Midwest and West and in suburban areas of the South. Additionally, Wilson et al. [7] found a direct association between the presence of TRI facilities in census tracts/blocks and high percentage non-White, and an inverse association between number of TRI facilities and high SES. The text *Toxic Wastes and Race in the United States* [14] demonstrates that extensive racial and socioeconomic disparities persist in the distribution of hazardous waste facilities and unhealthy land uses. Furthermore, Hutch et al. [15] found neighborhoods with hazardous waste facilities are populated by 56% people of color, whereas neighborhoods without hazardous waste facilities are occupied by only 30% people of color [15]. In metropolitan areas, where 80% of hazardous waste facilities are located, the residents in neighborhoods where these facilities are located are approximately 60% minority [15]. These results present a case for exploring the cumulative burden and impact of all toxic facilities in metro Atlanta and potential linkages to environmental health disparities.

We also found evidence that the capture and resolution of complaints of TRI facilities differ according to local demography. Minority communities—specifically census tracts with larger Black and Asian populations—were less likely to file complaints. It is possible that census tracts with higher minority populations may face barriers to filing complaints. They may lack the community capacity to file complaints, given the education and material resources necessary to navigate the complaint process. There may also be a history of past complaints not being resolved in a timely manner, which may discourage future complaints from being made. There is evidence of extended resolution time to complaints for census tracts with larger Hispanic populations in our dataset. This delayed timeline may be due to lack of enforcement, causing complaints to not be pursued as quickly in these communities. It is also possible that the complaints in these tracts may be a result of more serious issues and take longer to resolve for this reason.

Although no public health studies have investigated whether disparities exist with regard to complaints and resolution time for minorities and populations of low SES, there have been a few studies from other disciplines that have looked at regulation of air pollution. Konisky [16] found that for Hispanic communities, firms are not only more likely to be significant violators of their Clean Air Act obligations, but they are less likely to be designated as violators by regulatory offices. Hird [17] studied Superfund sites at the national and county level and concluded that more affluent communities were more likely to be represented in the Superfund cleanup program [17]. It was noted in that study that minorities are more likely to live in close proximity to hazardous sites, however these sites are less likely to be listed on the National Priorities List (NPL) [17].

This study has some limitations. We used 2006–2011 TRI data and 2000 census data, which we find to be appropriate, but may have introduced a small amount of misclassification. Additionally, we investigated the cumulative effect of TRI facility distribution and releases over a six-year period and we did not look at changes over time. These results provide a snapshot of burden disparities of TRI facilities in the Atlanta MSA. However, impacts on our findings would be minimal, because we expect that demographic changes of sufficient magnitude to be meaningful would take longer to occur. Future studies could examine this question with data covering more years. An additional limitation was the focus on facility location and releases but not the toxicity of the chemicals emitted. Previous research has shown that to understand burden and exposure disparities, it is important to examine toxicity of the chemicals emitted from the facilities [2]. There were also a limited number of variables with which to estimate community SES. More specifically, educational attainment was only available for females. Analysis of census data has shown that female educational attainment is a good representation of the overall educational attainment in census tracts, and that taking into consideration the educational attainment of all individuals living in the census tract would probably not change the results [18,19]. We assume that the complexity of complaints (and therefore, the time it would take to address them) did not differ between census tracts in a systematic way, although we were unable to test this assumption. We also make the assumption that complaints arose from the same census tract in which they were filed, which we find reasonable because the majority of the sources had releases of a magnitude that would primarily affect the local area.

## 5. Conclusions

This study’s findings contribute to the literature of exposure disparities by its focus on a major southern city, metropolitan Atlanta. It is one of the first to show that minority populations may find challenges in the complaint process. Specifically, census tracts with lower educational attainment and higher proportions of non-Whites are faced with the greatest burden of TRI facilities and potential health hazards. Even with a few methodological limitations, this study found statistically significant associations between census tract TRI distribution and education. Additionally, air toxic releases were inversely associated with educational attainment in a census tract. There is also evidence of differences of regulatory enforcement patterns related to air quality issues related to census-tract race-ethnicity. Complaints are less likely to be filed for TRI facilities with higher minority populations and take longer to resolve in others.

These results can be incorporated into the larger dialogue related to the equitable siting of hazardous facilities. Public policy should limit clustering of TRI facilities in minority and low-SES communities in both urban and rural areas. Finally, the complaint process could be reassessed to ensure that all residents have the opportunity to file complaints and that resolutions are made appropriately and quickly. Federal and state agencies can also improve enforcement of existing regulations to which facilities must adhere. In addition to government guidance and regulation, communities can utilize publicly-available data to educate themselves on potential environmental hazards and partner with institutions and agencies to investigate interventions and policy changes [15,20]. Information on complaints can be included in publically-available databases to provide a more transparent view of the TRI system.

## Figures and Tables

**Table 1:** Toxic Release Inventory (TRI) data (2006–2011) for Atlanta metropolitan statistical area (MSA) census tracts with TRI facilities (*N* = 135).

Variable	Mean	Std. Dev.	Min	Lower Quartile	Median	Upper Quartile	Max
Total number of air toxic releases	19.8	27.0	1.0	4.0	10.0	24.0	218.0
Total amount of air toxics emitted (lbs)	851,634.7	5,592,370.4	0	51.0	4055.0	59,137.9	5,715,497.1
Amount of air toxics emitted per release (lbs)	18,250.4	84,447.1	0	5.9	260.8	2566.5	742,272.4
Total number of complaints	2.9	4.0	1.0	1.0	2.0	3.0	33.0
Average time to resolve complaint (days)	36.4	65.7	0	1.0	5.0	44.6	386.0

**Table 2:** Socio-demographic characteristics of all census tracts within the 20 county Atlanta MSA (*N* = 676).

Variable	Mean	Std. Dev.	Min	Lower Quartile	Median	Upper Quartile	Max
Median Household Income (U.S. Dollars)	51,816	22,345	4705	37,223	48,081	63,075	163,474
% of population are female HS graduates	8.6	3.1	0	6.3	8.8	11.0	16.2
% of population are females with undergraduate degree	8.4	4.8	0	4.7	7.6	11.5	22.0
% of population are females with graduate degree	3.0	2.8	0	1.2	2.2	3.8	40.0
% of population White	56.1	32.7	0	23.7	68.2	85.7	100.0
% of population Black	32.7	33.2	0	6.3	16.8	57.0	100.0
% of population Hispanic	6.5	9.3	0	1.6	3.3	6.9	71.2
% of population Asian	2.9	3.6	0	0.4	1.7	3.8	20.5
% of households with income below the poverty line	11.4	11.3	0	4.3	7.8	13.9	75.4
% of households on public assistance	2.8	3.8	0	0.7	1.5	3.3	30.0
% of housing units vacant	5.6	4.4	0	3.1	4.7	6.6	57.9

**Table 3:** Bivariate associations of Toxic Release Inventory (TRI) facility presence.

Socio-Demographic Characteristics	Facility Present *N* = 135	No Facility Present *N* = 541	OR (95% CI)	*p* Value
Median Household Income				
>$77,500	6 (4.4%)	69 (12.8%)	1.00	
$50,000–$77,500	42 (31.1%)	197 (36.4%)	2.5 (1.0–6.0)	0.4
$22,500–$50,000	80 (59.3%)	234 (43.3%)	3.9 (1.6–9.4)	0.005
<$22,500	7(5.2%)	41 (7.5%)	2.0 (0.6–6.2)	0.85
% of population White, Median (IQR)	65.6 (44.0–83.4)	70.2 (21.6–87.0)	1.004 (0.998, 1.009)	0.2302
% of population non-White, Median (IQR)	32.5 (15.5–54.2)	27.9 (11.6–75.2)	0.997 (0.991, 1.002)	0.2616
% of population Black, Median (IQR)	17.5 (8.4–34.6)	16.1 (5.5–63.6)	0.993 (0.987, 0.998)	0.0240
% of population Hispanic, Median (IQR)	4.5 (2.0–10.9)	2.9 (1.3–5.9)	1.03 (1.013, 1.049)	0.0007
% of population Asian, Median (IQR)	0.9 (0.2–3.6)	1.6 (0.3–3.6)	0.992 (0.941, 1.044)	0.7517
% of Female HS diploma, Median (IQR)	9.6 (7.3–11.5)	8.5 (6.0–10.9)	1.115 (1.050, 1.183)	0.0009
% of Female Undergrad degree, Median (IQR)	5.7 (3.8–9.0)	8.3 (5.0–12.1)	0.904 (0.865, 0.944)	<0.0001
% of Female Grad degree, Median (IQR)	1.8 (0.9–2.7)	2.3 (1.2–3.9)	0.823 (0.745, 0.909)	0.0001
% of population with income below poverty line, Median (IQR)	10.7 (5.6–16.2)	7.7 (4.0–13.8)	1.002 (0.986, 1.018)	0.8119
% of Housing Vacant, Median (IQR)	5.3 (3.8–7.3)	4.7 (3.9–6.8)	1.017 (0.983, 1.052)	0.3396
% of Households on Public Assist, Median (IQR)	2.1 (1.0–3.7)	1.5 (0.7–3.3)	1.002 (0.955, 1.051)	0.1466

**Table 4:** Multivariate model results explaining TRI facility presence. OR: odds ratio.

Census Tract Characteristics	Unadjusted OR	Adjusted OR	*p* Value
Median Household Income			
>$77,500	1.00	1.0	
$50,000–$77,500	2.5 (1.0, 6.0)	1.7 (0.6, 4.3)	0.8
$22,500–$50,000	3.9 (1.6, 9.4)	2.4 (1.8, 6.7)	0.05
<$22,500	2.0 (0.6, 6.2)	1.7 (0.4, 8.0)	0.9
% Hispanic	1.03 (1.013, 1.049)	1.012 (0.994, 1.032)	0.2
% Black	0.993 (0.987, 0.998)	0.985 (0.977, 0.993)	0.0005
% females with undergrad degree	0.904 (0.865, 0.944)	0.894 (0.842, 0.949)	0.0002

**Table 5:** Multivariate model results explaining TRI chemical releases.

Census Tract Characteristics	Total Amount Toxic Air Chemicals Released		Average Amount of Toxic Air Chemicals per Release		Number of Toxic Air Chemical Releases	
	Slope Coefficient (95% CI)	*p* Value	Slope Coefficient (95% CI)	*p* Value	Slope Coefficient (95% CI)	*p* Value
Median Household Income	−0.0000082 (−0.0005, 0.0003)	0.62	0.000011 (−0.004, 0.006)	0.80	−0.00022 (−0.00069, 0.00026)	0.37
% Females with undergrad degree	−0.19 (−0.13, −0.24)	0.009	−0.18 (−0.11, −0.22)	0.004	0.96 (−0.74, 2.66)	0.26
% Non-White	0.0080 (−0.067, 0.09)	0.35	0.0061 (−0.07, 0.89)	0.37	0.0035 (−0.20, 0.20)	0.97

Note: total amount of chemicals emitted, amount of chemicals emitted per release, percent of population female with a undergraduate degree, percent of population Hispanic, and percent of population Black were LOG10 transformed.

**Table 6:** Multivariate models explaining complaint data.

Census Tract Characteristics	Number of Complaints		Time to Resolution of Complaints	
	Adjusted OR	*p* Value	Adjusted OR	*p* Value
TRI Facility				
Present (1)	4.065 (2.779, 5.935)	<0.0001	1.723 (1.100, 2.732)	0.02
Absent (0)	1.00		1.00	
Median Household Income	1.000 (1.000, 1.000)	0.44	1.000 (1.000, 1.000)	0.78
% of population Black	0.989 (0.983, 0.995)	0.0006	0.994 (0.985, 1.003)	0.20
% of population Hispanic	1.020 (1.000, 1.036)	0.05	1.031 (1.010, 1.054)	0.0101
% of population Asian	0.922 (0.875, 0.971)	0.0021	-	-
% of population female undergrad degree	0.957 (0.929, 1.021)	0.05	1.007 (0.941, 1.078)	0.83
